# Outpatient Initiation of Sotalol: Reappraising Risk, Monitoring, and Modern Care Pathways

**DOI:** 10.31083/RCM52301

**Published:** 2026-10-21

**Authors:** Rakesh Agarwal, Dimitrios Lypourlis

**Affiliations:** ^1^Department of Cardiology, Lyell McEwin Hospital, Elizabeth Vale, SA 5112, Australia; ^2^College of Health, Adelaide University, Adelaide, SA 5000, Australia

**Keywords:** sotalol, torsades de pointes, QT, atrial fibrillation

## Abstract

Sotalol remains an important antiarrhythmic drug for rhythm control in atrial fibrillation and other supraventricular arrhythmias. However, sotalol is traditionally initiated in the inpatient setting because of concerns regarding QT prolongation and torsades de pointes. This practice is based on early observational data collected before the availability of contemporary risk stratification, standardized protocols, and modern rhythm monitoring technologies. Over the past two decades, evidence from observational studies using device-based monitoring, protocolized outpatient clinics, and remote electrocardiographic technologies has demonstrated low rates of malignant ventricular arrhythmias in carefully selected patients. These data also highlight the importance of longitudinal surveillance, as clinically relevant QT prolongation may occur beyond the conventional inpatient monitoring window. Thus, this review examines the pharmacologic basis of sotalol-related proarrhythmia, critically reassesses the rationale for inpatient initiation, synthesizes contemporary evidence supporting outpatient strategies in carefully selected patients with protocolized monitoring, and proposes a practical, risk-stratified framework for outpatient sotalol initiation while identifying key knowledge gaps that still require prospective validation.

## 1. Introduction

Rhythm control has been associated with greater freedom from atrial fibrillation (AF), a lower incidence of atrial cardiomyopathy, and improved cardiovascular and cerebrovascular outcomes [1,2,3]. Commonly used antiarrhythmic drugs (AADs) for rhythm control in AF include class Ia, Ic, and III agents. Sotalol, a class III AAD, is widely used to manage AF and atrial and ventricular arrhythmias and is readily available. In Europe and the United States of America (US), sotalol is the second most common AAD, after amiodarone, and is used for AF in individuals with structural heart disease (SHD); sotalol is also frequently used (~13%) in individuals with no or minimal structural heart disease [4]. In Australia, sotalol is one of three available AADs (flecainide and amiodarone) for rhythm control in AF.

Despite widespread use, initiation of sotalol has long been constrained by concerns regarding proarrhythmia, particularly QT interval prolongation and a 1–4% risk of torsades de pointes (TdP), leading to the conventional requirement for inpatient initiation with continuous electrocardiographic (ECG) monitoring [5,6,7]. Sotalol continues to carry a regulatory black-box warning recommending initiation in a facility capable of continuous ECG monitoring and cardiac resuscitation for at least three days [8]. The American College of Cardiology (ACC)/American Heart Association (AHA) guidelines recommend additional measurement of serum potassium and magnesium during initiation [9]. The European Society of Cardiology (ESC) guidelines suggest ECG monitoring at baseline, after 1 day, and after 1–2 weeks in patients on sotalol [10]. This paradigm was shaped by early observational studies that reported frequent dose-limiting events during initiation, despite malignant ventricular arrhythmias being uncommon [11,12].

Over the past two decades, a growing body of evidence suggests that outpatient initiation of sotalol may be safe in selected patient cohorts [13,14,15]. More than 50% of physicians from Europe and the US in the Antiarrhythmic Medication for AF (AIM-AF) trial reported outpatient loading of sotalol [16]. Observational data indicate that clinically meaningful adverse events are driven primarily by bradyarrhythmias and QT prolongation, and that these risks may persist beyond the traditional 72-hour inpatient monitoring window [17,18,19]. Preliminary data from small observational studies also suggest safety in the pediatric population [20,21]. With the increasing use of cardiac implantable electronic devices (CIEDs) with remote monitoring capabilities and access to ambulatory rhythm monitoring technologies, including wearables, outpatient initiation of sotalol has emerged as a clinically relevant and increasingly common practice, although mechanistic guidance remains limited [14,22,23,24]. Meanwhile, hospitalization costs have risen rapidly, and there has been an increased emphasis on value-based, patient-centered care, and outpatient sotalol may offer a viable solution.

In this narrative review, we reassess the rationale for initiating inpatient sotalol therapy in the context of contemporary pharmacokinetic and electrophysiologic understanding, and propose a pragmatic, risk-stratified approach to outpatient sotalol initiation based on careful patient selection, standardized QT assessment, and ongoing surveillance. For this review, PubMed and Scopus databases were searched from inception using MeSH and non-MeSH generic terms, and studies reporting on outpatient initiation of sotalol were included (Table 1).

**Table 1.** **Search strategy for narrative synthesis**.

PubMed search (“sotalol”[MeSH Terms] OR “sotalol”[Title/Abstract]) AND (“outpatient”[Title/Abstract] OR “ambulatory”[Title/Abstract] OR “home”[Title/Abstract] OR “remote”[Title/Abstract] OR “clinic”[Title/Abstract]) AND (“initiat*”[Title/Abstract] OR “load*”[Title/Abstract] OR “commenc*”[Title/Abstract])
Scopus search TITLE-ABS-KEY (sotalol) AND TITLE-ABS-KEY (outpatient OR ambulatory OR home OR remote OR clinic) AND TITLE-ABS-KEY (initia* OR load* OR commenc*)

## 2. Pharmacology and Mechanisms of Proarrhythmia

Sotalol has a relatively predictable pharmacokinetic profile. Furthermore, sotalol has an oral bioavailability of nearly 100%, reaches peak serum levels in approximately 2–3 hours, exhibits minimal blood–brain barrier penetration, and is primarily excreted through the kidneys, with a half-life of 10–20 hours [25,26]. However, renal function is dynamic, particularly in older patients and those with even modest kidney dysfunction [27]. Additionally, steady-state plasma concentrations may not be achieved in these patients by discharge, even after five standard doses [28].

Sotalol exhibits dual electrophysiologic effects: nonselective β-adrenergic blockade and inhibition of the rapid component of the delayed rectifier potassium current (IKr). Sotalol is a racemic mixture of d- and l-isomers, and its mechanism of action reflects the properties of these isomers. The d-isomer of sotalol exhibits class III antiarrhythmic effects by acting on IKr. This leads to prolonged action potential duration (APD) and refractory period, manifesting as a prolonged QT interval on the surface ECG. The l-isomer, while prolonging repolarization, has nonselective β-blockade properties. β-blockade contributes to heart rate control and suppression of adrenergically mediated triggers.

A defining electrophysiologic feature of sotalol is reverse use dependence, in which potassium channel blockade and QT prolongation intensify at slower heart rates. This increases susceptibility to pause-dependent early afterdepolarizations and polymorphic ventricular arrhythmias, particularly during bradycardia or prolonged pauses [29,30]. Owing to its electrophysiologic profile, sotalol is used primarily to maintain sinus rhythm rather than for acute pharmacological cardioversion of AF.

Women appear more susceptible to sotalol-induced TdP. Compared with men, women have a longer baseline QT interval after puberty and a reduced repolarization reserve, driven in part by lower expression of repolarizing potassium currents, particularly IKr—the principal target of the class III effect of sotalol [31]. These intrinsic differences are compounded by the reverse use dependence of sotalol, as women are more prone to bradycardia during β-blockade, which amplifies QT prolongation at slower heart rates [32,33,34].

Electrolyte abnormalities, particularly hypokalemia and hypomagnesemia, substantially increase susceptibility to sotalol-related QT prolongation and TdP, even at relatively low doses. These abnormalities reduce repolarization reserve and promote early afterdepolarizations during IKr blockade [35]. Therefore, proarrhythmic risk with sotalol is multifactorial, influenced by baseline QT interval, renal function, heart rate, electrolyte balance, sex, and concomitant QT-prolonging medications. Heart rate, autonomic tone, electrolyte balance, renal function, and drug interactions evolve dynamically and influence repolarization reserve; thus, QT prolongation may emerge or worsen weeks to months after initiation, even in patients who tolerated early dosing without incident.

Hence, strategies that prioritize heart rate stabilization, standardized QT assessment, and close long-term rhythm surveillance—whether delivered in the inpatient or outpatient setting—are more closely aligned with the biology underlying sotalol-related proarrhythmia (Fig. 1). From this perspective, the central question is not whether sotalol is initiated inside or outside the hospital, but whether monitoring is deployed in a manner that reflects how pharmacologic risk actually evolves.

**Fig. 1.**
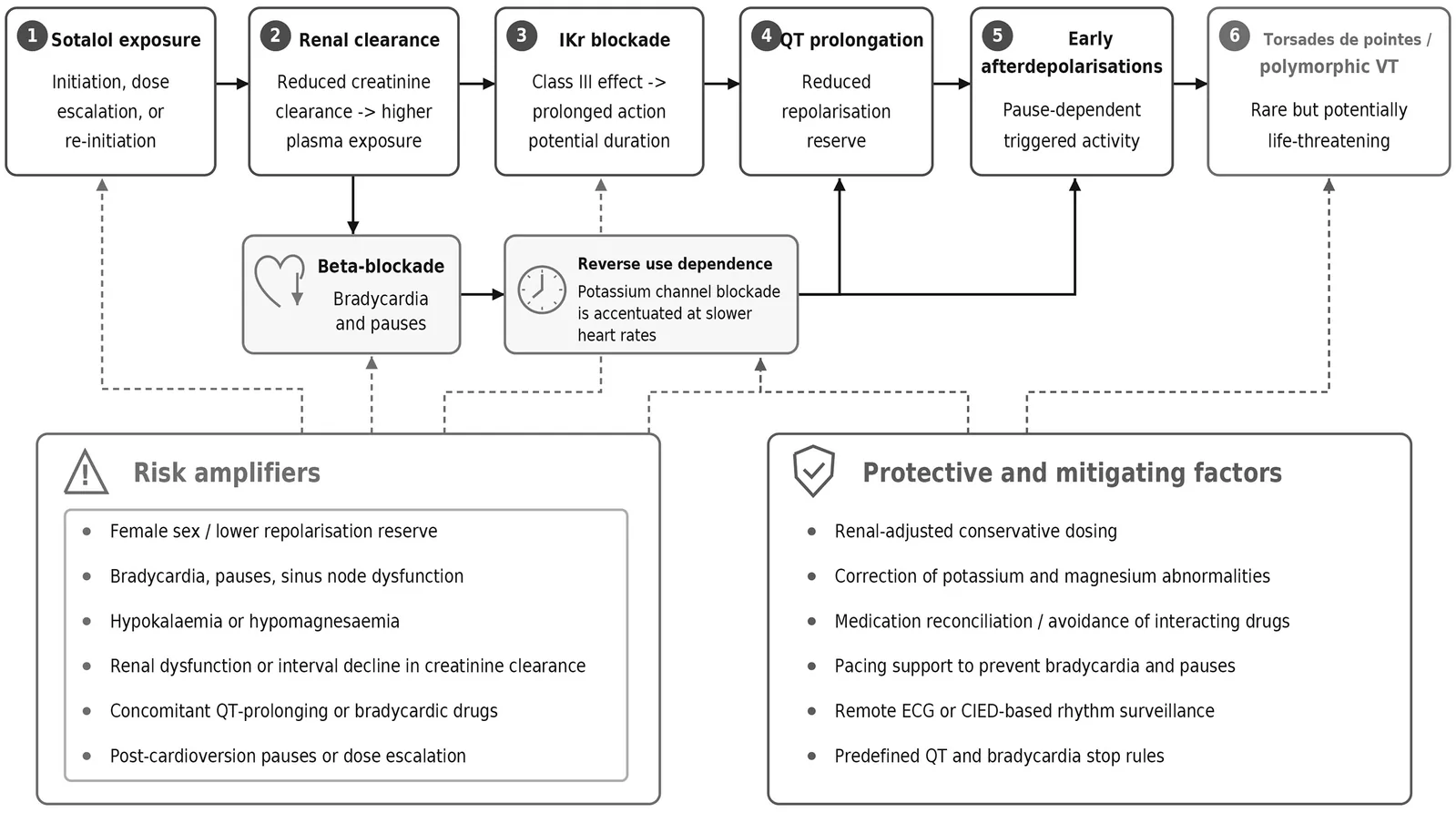
**Mechanistic pathways of sotalol-related proarrhythmia**. ECG, electrocardiogram; VT, ventricular tachycardia; CIED, cardiac implantable electronic device; IKr, rapid component of the delayed rectifier potassium current.

## 3. Historical Origins of Routine Inpatient Monitoring

The practice of routine inpatient sotalol initiation was largely established by early observational studies demonstrating frequent clinically significant events during loading. Soyka et al. [11] reported a 4.3% incidence of proarrhythmia with sotalol, including a ~1.9% incidence of TdP. Chung et al. [19] demonstrated that 1 in 5 patients experienced significant complications during inpatient initiation, driven primarily by bradyarrhythmias and QT prolongation, with TdP occurring in 5.8% of cases. Notably, 22 of the 25 cases with complications were detected within the first three days of initiation. Baseline ECG parameters and the absence of structural heart disease failed to identify a low-risk subgroup, reinforcing a conservative approach.

Subsequent inpatient series, including comparative analyses of sotalol and dofetilide initiation, confirmed that adverse events during initiation were common but rarely catastrophic [18].

## 4. What Inpatient Monitoring Actually Accomplishes

A critical reappraisal of inpatient initiation reveals that hospitalization primarily facilitates early detection of QT prolongation and bradyarrhythmias rather than preventing malignant ventricular arrhythmias. Most clinically actionable events involve asymptomatic QT prolongation or sinus node dysfunction, prompting dose adjustment. Prospective ambulatory monitoring studies have demonstrated that many clinically relevant adverse events occur beyond the conventional 72-hour inpatient window, challenging the adequacy of brief hospitalization for monitoring [15,17].

## 5. Regulatory, Guideline and Medicolegal Considerations

The regulatory and guideline landscape surrounding sotalol initiation remains complex. Sotalol continues to carry a black-box warning recommending initiation in a facility capable of continuous electrocardiographic monitoring and cardiac resuscitation for at least three days because of the risk of QT prolongation and TdP. Similarly, contemporary ACC/AHA and ESC guidance continues to support careful ECG and electrolyte monitoring during initiation, particularly in patients at elevated proarrhythmic risk.

Meanwhile, contemporary real-world practice patterns increasingly include outpatient initiation of sotalol for selected patients, particularly in specialized electrophysiology programs that use protocolized monitoring pathways. This creates an important tension between evolving clinical practice and traditional regulatory labeling. Accordingly, outpatient initiation should not be interpreted as a universally guideline-endorsed replacement for inpatient monitoring, but rather as an emerging and pragmatic strategy that may be appropriate for carefully selected patients when implemented within structured surveillance frameworks.

Therefore, implementation of outpatient pathways should include careful patient selection, explicit documentation of risk-benefit discussions, clear institutional protocols, predefined stop rules, and reliable access to longitudinal ECG and clinical surveillance. Shared decision-making remains essential, particularly for higher-risk individuals or in settings with limited remote monitoring infrastructure.

## 6. Evidence for Outpatient Sotalol Initiation

Several observational studies have reported safe outpatient initiation of sotalol with conservative dosing, adequate surveillance, and close monitoring (Table 2A and 2B, Ref. [14,15,17,36,37]).

**Table 2A.** **Study characteristics and patient selection in outpatient sotalol initiation studies**.

Study	Study design/setting	Country/center	Sample size	Rhythm indication	Starting dose/dosing strategy	Baseline QTc criteria	Renal function criteria	Key exclusion criteria	Key selection considerations
Kamsani et al., 2024 [15]	Observational cohort; outpatient arrhythmia clinic pathway	Australia; outpatient arrhythmia clinics	880	Atrial fibrillation and ventricular arrhythmia	80 mg twice daily in 53.2%	Baseline QTc 431 ± 32 ms; excessive QTc prolongation defined as QTc >500 ms or >25% increase	N/A	Non-completion of 1 month review; sotalol used on as-required basis only	Large contemporary outpatient cohort; likely selected patients suitable for serial outpatient ECG surveillance
Mascarenhas et al., 2021 [14]	Observational cohort; device-supported outpatient initiation	USA	105	Atrial fibrillation	40–80 mg twice daily	QTc >450 ms excluded	Creatinine clearance <40 mL/min excluded	LVEF ≤55%, CrCl <40 mL/min, QTc >450 ms, concomitant antiarrhythmic drugs, bradycardia, cognitive dysfunction	Highly selected cohort with preserved LVEF and implanted rhythm monitoring devices; pacing support used to mitigate bradycardia
LaBreck et al., 2025 [36]	Pharmacist-led outpatient antiarrhythmic clinic pathway	USA; outpatient antiarrhythmic clinic	263	Atrial fibrillation and ventricular arrhythmias	Median starting dose 120 mg twice daily; 80 mg twice daily if CrCl <60 mL/min; final dose unchanged in 82.5%	Baseline QTc <450 ms, or <500 ms with intraventricular conduction delay	Dose adjusted for CrCl <60 mL/min	Pregnancy, CrCl <30 mL/min, LVEF <45%, history of TdP, concomitant β-blocker use without pacing support, unexplained syncope, Ami within 30 days, class IV heart failure	Structured pharmacist-led pathway requiring remote ECG capability and protocol adherence
Hauser et al., 2003 [17]	Structured outpatient clinical pathway using transtelephonic monitoring	USA	Approximately 37 patients receiving sotalol	Atrial fibrillation or atrial flutter	Physician-directed dosing	N/A	N/A	Patients with permanent pacemakers excluded from bradycardia endpoint analysis	Early feasibility experience; small sample size and limited contemporary generalizability
Roberts et al., 2022 [37]	Physician–pharmacist outpatient loading program/quality-improvement style evaluation	USA; arrhythmia clinic	12	Atrial fibrillation	Four-step process with physician referral, a pharmacist educational visit, a 3-day load, and a wrap-up visit	Baseline QT <450 ms	CrCl <50 mL/min excluded	Pregnancy, CrCl <50 mL/min (adjusted body weight), LVEF <45%, presence of ventricular arrhythmias, tremor, concurrent use of any QTc prolonging medication, and known medical noncompliance	Supports feasibility and cost reduction

CrCl, creatinine clearance; LVEF, left ventricular ejection fraction; ms, milliseconds; N/A, not available; TdP, torsades de pointes; QTc, corrected QT interval.

**Table 2B.** **Monitoring protocols, safety outcomes, and limitations in outpatient sotalol initiation studies**.

Study	ECG/rhythm monitoring schedule	QTc correction method	Dose adjustment/stop rules	Follow-up duration	QT prolongation/dose-limiting events	TdP/ventricular arrhythmia/death	Key limitations
Kamsani et al., 2024 [15]	12-lead ECG at baseline, day 3, day 7, 1 month, then as clinically required	Bazett formula; modified QTc formula used for bundle branch block or ventricular pacing	Dose reduction or cessation for QTc >500 ms or >25% increase from baseline; higher starting dose predicted QT prolongation	Minimum 1 month; ongoing ECGs thereafter	QT prolongation in 4 patients within 3 days, 1 within 1 week, and 5 after 1 month; late QT prolongation also reported at day 60 and very late follow-up	No TdP, ventricular arrhythmia, syncope, or sotalol-related death in the first month	Observational design; selected outpatient population; rare events underpowered; late QT prolongation highlights need for longitudinal surveillance
Mascarenhas et al., 2021 [14]	QT assessed using intracardiac electrograms and 12-lead ECGs; manual transmission 2 hours post-dose; daily ECGs for 3 days, weekly for 2 weeks, then at 1 month	N/A	Bradycardia mitigated by atrial pacing at lower rate of 70 bpm; QT monitored during initiation	Mean 23 ± 15 months	35 patients discontinued sotalol, out of which 4 had bradycardia	No QT-related mortality or TdP; 66% successfully maintained on sotalol	Highly selected device population; preserved LVEF; excluded renal dysfunction, bradycardia, cognitive dysfunction, and baseline QT prolongation; findings may not apply to non-device patients
LaBreck et al., 2025 [36]	Kardia Mobile 6L remote ECG; uploads twice daily for 3 days; ECG 2 hours post-dose; baseline labs and 12-lead ECG reviewed	Bazett formula	Pharmacist review; physician notified if QTc ≥500 ms or atrial fibrillation; dose decreased if QTc >500 ms, or >550 ms in ICD/intraventricular conduction delay	Minimum 90 days	98.9% completed 3-day load; 6.5% stopped due to bradycardia	No TdP; 1 death over 90 days, not QT-related	Requires patient access to remote ECG technology, digital literacy, and adherence; observational programmatic design; rare arrhythmic events remain difficult to exclude
Hauser et al., 2003 [17]	30-second ECG once daily using transtelephonic monitoring	N/A	Physician-driven dose adjustment	Several weeks	None	Demonstrated outpatient feasibility; specific rare event estimates limited	Small early cohort; older monitoring technology; limited detail on QT thresholds, renal criteria, and exclusion criteria
Roberts et al., 2022 [37]	Physician–pharmacist monitoring program; details NR in current manuscript table	Bazett formula	Physician–pharmacist monitoring	3-day loading phase	Fatigue (4, 33.3%) and bradycardia (3, 25%)	None	Primarily supports feasibility and cost comparison; limited detailed safety-event reporting in current manuscript table

ECG, electrocardiogram; ICD, implantable cardioverter defibrillator; LVEF, left ventricular ejection fraction; TdP, torsades de pointes; NR, not reported; bpm, beats per minute.

## 7. Device-Supported Outpatient Initiation

Outpatient initiation in patients with CIEDs is among the most clinically and mechanistically compelling alternatives to inpatient loading. Continuous rhythm surveillance with the existing CIED, along with pacing support, directly mitigates bradycardia-mediated proarrhythmia. Mascarenhas et al. [14] reported no QT-related adverse events or TdP during a 23-month follow-up in a carefully selected cohort of 105 patients with implanted pacemakers, implantable cardioverter defibrillators, or loop recorders who initiated sotalol at standard doses. CIEDs were programmed to pace at a lower atrial rate of 70 bpm [14]. These findings underscore the protective role of heart rate stabilization and continuous monitoring in the contemporary era of digital devices.

## 8. Protocolized Clinic-Based Outpatient Initiation

Large contemporary cohorts have evaluated protocolized outpatient clinic-based initiation strategies that include a scheduled ECG follow-up. In the largest outpatient series published to date, early QT prolongation necessitating dose modification was uncommon, occurring in only 4 of 880 patients within 3 days and in 1 patient within the first week [15]. No ventricular arrhythmias, syncope, or sotalol-related mortality were observed during the initial treatment phase. Importantly, a small but meaningful incidence of late QT prolongation was identified, with five patients developing late QT prolongation. One patient developed QT prolongation after two months, while one developed QT prolongation after 1423 days, emphasizing the need for continued surveillance beyond initiation.

## 9. Remote and Home-Based ECG-Guided Initiation

Advances in digital health have enabled home-based initiation strategies using validated remote electrocardiographic technologies. Pharmacist-led outpatient programs employing handheld (Kardia Mobile 6L) or smartphone-enabled ECG devices have demonstrated safe outcomes and high protocol adherence. LaBreck et al. [36] showed the safety of a pharmacist-led protocol where participants monitored their QTc using the Kardia Mobile 6L device. Smaller quality-improvement studies integrating physician–pharmacist collaboration further support the feasibility, safety, and cost-effectiveness of these approaches. Roberts et al. [37] reported an outpatient loading cost of USD 886.30, compared with USD 7571.76 for inpatient loading.

## 10. Patient Selection: Who Is (and Is Not) Appropriate for Outpatient Initiation

Across studies, safe outpatient initiation depends critically on careful patient selection. Favorable characteristics include a baseline QTc below accepted thresholds (with appropriate adjustment in the presence of conduction delay), preserved renal function, stable electrolyte levels, and absence of significant baseline bradycardia. Patients with permanent pacing, continuous rhythm monitoring capabilities, or those willing to undergo routine and scheduled ECG monitoring appear particularly well suited for outpatient initiation.

Female sex, advanced age, renal impairment, and concomitant QT-prolonging medications warrant heightened caution. Individuals with advanced conduction disease without pacing support, marked sinus node dysfunction, prior TdP, or severe renal dysfunction are best managed with inpatient strategies. Current evidence supports a risk-stratified approach rather than indiscriminate outpatient initiation.

## 11. A Practical Framework for Outpatient Sotalol Initiation

A pragmatic outpatient initiation framework should include:

(1) Pre-initiation assessment: baseline ECG, renal function, electrolyte evaluation, and medication review.

(2) Standardized dosing: conservative initial dosing with renal adjustment.

(3) Structured monitoring: scheduled ECG assessments during early therapy and beyond the initiation phase.

(4) Explicit stop rules: predefined QT and heart-rate thresholds prompting dose reduction or discontinuation.

Integration of remote ECG technologies and pharmacist-led monitoring can enhance adherence and early detection of adverse effects.

## 12. Risk-Stratified Framework for Outpatient Sotalol Initiation

When implemented, outpatient initiation pathways should be delivered through an institutionally approved protocol that includes explicit eligibility criteria, renal-adjusted dosing guidance, standardized QT assessment, scheduled surveillance, predefined stop rules, and escalation pathways. The proposed thresholds and operational pathways are derived largely from observational experience and expert interpretation rather than prospective randomized validation.

All patients considered for outpatient sotalol initiation should undergo a structured baseline eligibility assessment, including QTc evaluation, CrCl, electrolyte assessment (serum potassium and magnesium), medication reconciliation, assessment of resting heart rate and pauses, screening for conduction disease, and assessment of ability to comply with follow-up. Patients with few or no elevated-risk features may undergo standard outpatient initiation. In contrast, those with multiple risk modifiers should receive enhanced monitoring with remote ECG or continuous device-based surveillance. Patients with marked baseline bradycardia (resting heart rate <50 bpm), pauses ≥3 seconds, advanced conduction disease without pacing support, prior TdP, severe renal dysfunction, marked baseline QT prolongation, decompensated heart failure, unstable ischemia, or inability to comply with structured ECG surveillance should generally be excluded from outpatient initiation pathways.

Baseline QT assessment should account for underlying rhythm and conduction abnormalities. In patients with AF, QT measurement should ideally be averaged over multiple cardiac cycles to minimize beat-to-beat variability. In the presence of bundle branch block, ventricular pacing, or wide QRS complexes, modified QT correction approaches should be considered to avoid overestimating repolarization duration. Sex-specific thresholds should be incorporated into patient selection, with QTc ≤450 ms in men and ≤470 ms in women generally considered acceptable for outpatient initiation in the absence of other major risk modifiers.

Initial dosing should remain conservative and renal-adjusted, with 80 mg twice daily typically reserved for patients with preserved renal function and lower-risk profiles. Patients with moderate renal impairment or borderline bradycardia may require lower or once-daily dosing strategies. Repeat ECG surveillance should occur following dose escalation, significant changes in renal function, electrolyte abnormalities, interruption and re-initiation of therapy, or electrical cardioversion, because pause-dependent QT prolongation may emerge during these periods. Mandatory QT surveillance should include ECG at baseline, day 3, day 7, and one month, with more frequent early monitoring using remote or device-based ECG in higher-risk patients. Re-initiation after prolonged interruption or dose escalation should be approached similarly to *de novo* initiation, particularly in patients with interval changes in renal function or concomitant medications.

Structured stop rules should include QTc ≥500 ms, a ≥25% increase from baseline QTc, symptomatic bradycardia, pauses ≥3 seconds, new ventricular arrhythmias, or clinically significant deterioration in renal function. Longitudinal ECG and renal surveillance remain essential because proarrhythmic risk evolves dynamically over time (Fig. 2).

**Fig. 2.**
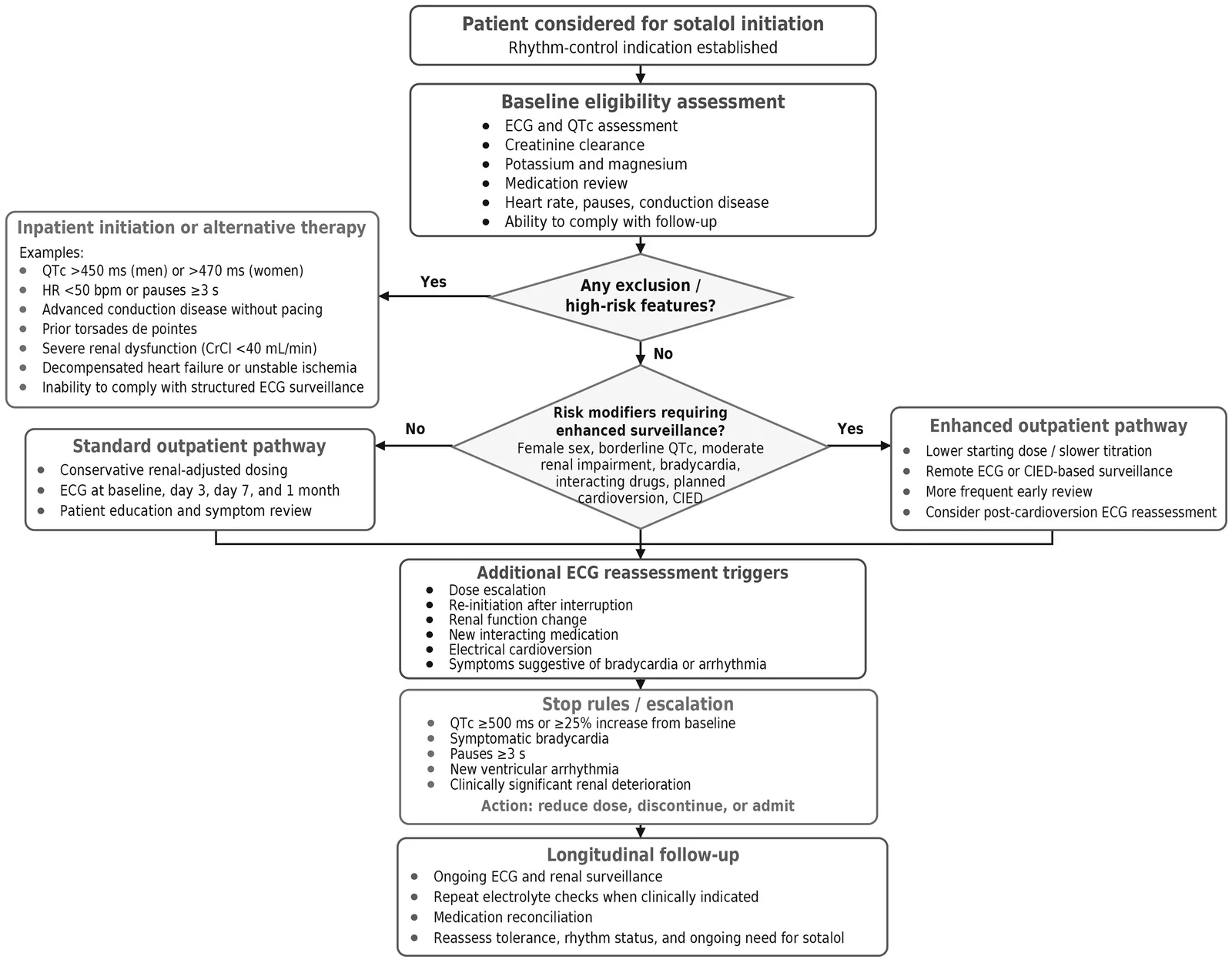
**Risk-stratified framework for outpatient sotalol initiation**. HR, heart rate; CIED, cardiac implantable electronic device; CrCl, creatinine clearance; QTc, corrected QT interval.

## 13. Discussion

The current evidence base supports reassessing how sotalol initiation is conceptualized, but it does not eliminate the need for caution. Historically, inpatient telemetry was adopted as a pragmatic response to early reports of proarrhythmia during drug loading. However, a deeper understanding of sotalol pharmacokinetics and electrophysiology suggests that proarrhythmic risk extends beyond the first several inpatient doses. Sotalol-related risk is dynamic and influenced by heart rate, renal function, electrolyte balance, autonomic tone, sex, and concomitant QT-prolonging or bradycardic medications. Therefore, safe initiation of sotalol depends not only on the setting but also on structured, sustained monitoring.

Available outpatient studies suggest that sotalol can be initiated safely in carefully selected patients with conservative dosing, standardized QT assessment, and structured rhythm surveillance. However, these findings derive primarily from observational cohorts and should not be interpreted as establishing equivalence or non-inferiority to inpatient initiation. Device-supported pathways further underscore this principle: continuous pacing and remote monitoring directly mitigate bradycardia-mediated risk in a manner that telemetry alone cannot. Similarly, remote ECG–guided and pharmacist-led programs show that high-frequency early monitoring, coupled with longitudinal follow-up, identifies clinically relevant QT changes beyond the traditional hospitalization window [14,36,37]. The risk-stratified approach we outline is directly informed by contemporary outpatient and device-supported sotalol cohorts. In the largest outpatient series, Kamsani et al. [15] demonstrated that conservative initial dosing and scheduled ECG surveillance on days 3, 7, and 1 month were associated with no ventricular arrhythmias or sotalol-related mortality, while identifying late QT prolongation that would not have been captured by short inpatient observation. LaBreck et al. [36] further showed that high-frequency remote ECG monitoring during the early loading phase allows timely dose adjustment using standardized QT thresholds, with high protocol adherence and no excess proarrhythmia. In patients with cardiac implantable electronic devices, Mascarenhas et al. [14] reported the complete absence of TdP or QT-related death, underscoring the protective role of heart-rate stabilization and continuous rhythm surveillance. Together, these studies support a model in which sotalol safety is governed by rate control, QT reserve, and longitudinal monitoring rather than by the clinical setting where the drug is initiated.

Routine inpatient initiation of sotalol imposes substantial costs, driven largely by hospital room utilization rather than medication-related expenses. Outpatient initiation strategies consistently yield substantial cost savings while improving patient convenience and access [38,39].

Initiating sotalol as a routine practice in outpatient settings poses practical barriers. Ensuring reliable patient adherence to follow-up, including ECG acquisition and regular laboratory testing, can be challenging. A remote ECG strategy depends on access to validated devices, digital literacy, and clinical capacity to review transmissions promptly. These requirements limit generalizability for patients with limited health literacy, cognitive impairment, socioeconomic barriers, and reduced ability to attend serial monitoring visits.

There are also important workflow, governance, and medicolegal considerations. Outpatient initiation should ideally be delivered through an institutionally approved protocol that includes explicit eligibility criteria, renal-adjusted dosing guidance, standardized QT measurement methods, predefined stop rules, documentation of shared decision-making, and clear escalation pathways. Clinicians should also document the rationale for outpatient initiation, the discussion of alternatives, including inpatient monitoring, and the understanding of warning symptoms and monitoring requirements by patients. These safeguards are particularly important because regulatory labeling and guideline recommendations remain more conservative than some contemporary real-world practice patterns.

The current evidence base supporting outpatient initiation of sotalol remains limited by the observational design of available studies, a lack of randomized data, highly selected patient populations, heterogeneous monitoring strategies, and low overall event rates. Most published cohorts were conducted within structured clinical pathways that incorporate intensive follow-up and protocolized surveillance, which may not be generalizable to broader practice settings. Furthermore, the rarity of TdP limits the ability of existing studies to exclude clinically meaningful differences in risk compared with inpatient initiation. Late QT prolongation has emerged as a consistent signal across outpatient cohorts, reinforcing the need for ongoing ECG surveillance regardless of initiation setting. Future research should move beyond retrospective feasibility and focus on prospective comparative studies.

## 14. Conclusions

Contemporary observational evidence suggests that outpatient initiation of sotalol may be feasible for carefully selected patients when paired with protocolized monitoring and longitudinal surveillance. However, current evidence does not establish equivalence to traditional inpatient initiation strategies, and prospective comparative studies remain necessary. Outpatient initiation should be viewed as a potentially feasible, risk-stratified strategy for selected patients rather than a proven alternative to inpatient hospitalization. Ongoing surveillance and individualized clinical judgment remain essential to minimize proarrhythmic risk.
